# Biopsies from Idiopathic Normal Pressure Hydrocephalus Patients as a Window to the Events of Early Alzheimer's disease: Beta‐amyloid and Tau Pathologies Alter Synaptic Transmission in Human Brain

**DOI:** 10.1002/alz70855_097565

**Published:** 2025-12-23

**Authors:** Antonios Dougalis, Polina Abushik, Anssi Pelkonen, Luca Giudice, Mireia Gomez‐Budia, Nataliia Novosolova, Nelli‐Noora Välimäki, Mohammad Rezaie, Dilyara Nurkhametova, Raisa Giniatullina, Anastasia Shakirzyanova, Akash Mali, Christiaan PJ de Kock, Eline J Mertens, Huibert D Mansvelder, Beth Stevens, Evan Macosko, Mikko Hiltunen, Tuomas Rauramaa, Ville Leinonen, Tarja Malm

**Affiliations:** ^1^ University of Eastern Finland, Kuopio, Finland; ^2^ Vrije Universiteit Amsterdam, Amsterdam, Netherlands; ^3^ Broad Institute of MIT and Harvard, Cambridge, MA, USA; ^4^ Boston Children's Hospital, Boston, MA, USA; ^5^ Kuopio University Hospital, Kuopio, Finland

## Abstract

**Background:**

The molecular mechanisms leading to Alzheimer's disease (AD) are poorly known. This is due to the lack of human tissue samples for research representing early changes of AD pathology, raising a need for development of novel human‐based preclinical models for AD. Idiopathic normal pressure hydrocephalus (iNPH) is an neurodegenerative disease characterized by an impaired cerebrospinal fluid (CSF) clearance. iNPH is treated by a shunt surger to drain the excess CSF into the abdominal cavity. During the surgery, 10‐20mm^3^ Broadman area 8 biopsy can be excised by minimally invasive methods for preclinical studies. Due to the early AD‐related pathology present in a subpopulation of iNPH patients, the brains of these patients offer a unique window to evaluate cellular events taking place during the course of AD pathology progression.

**Method:**

We have set up a pipeline to evaluate, in a layer and cell‐type dependent manner, the intrinsic neuronal operational properties in the iNPH biopsies. Our patch clamp and multi electrode array (MEA) studies show that these biopsies are viable and retain the required microcircuit to study network and synaptic function in human cortex. We have carried out integrative analysis of human neuronal electrophysiology at single neuron and network level followed by subsequent cellular morphological reconstructions to register the primary pathological changes in neuronal functions in correlation with existing AD‐related pathology.

**Result:**

The presence of Aβ deposits induced a decrease in the L1‐induced inhibition and led to hyperexcitability in response to application of NMDA in MEA recordings. Interestingly, the global spine density of supraganular pyramidal neurons was increased in biopsies with AD‐related pathology. Pyramidal neurons in cases with both Aβ and tau exhibited more consistent deficits in the intrinsic neuronal properties with increase in sodium and potassium currents and a strong propensity to bursting under NMDA stimulation.

**Conclusion:**

This is the first study to show that the accumulation of Aβ and tau alters synaptic transmission and consolidation of a hyperexcitable supragranular cortical network. The iNPH biopsies provide a unique opportunity to unravel how AD‐related pathology alters neuronal network functionality in humans.

